# Supplementary figures and images for: A recombinase-activated ribozyme to knock down endogenous gene expression in zebrafish
Source: PLoS Genet. 2025 Feb 7;21(2):e1011594. doi: 10.1371/journal.pgen.1011594 (PMC11856399; doi:10.1371/journal.pgen.1011594)

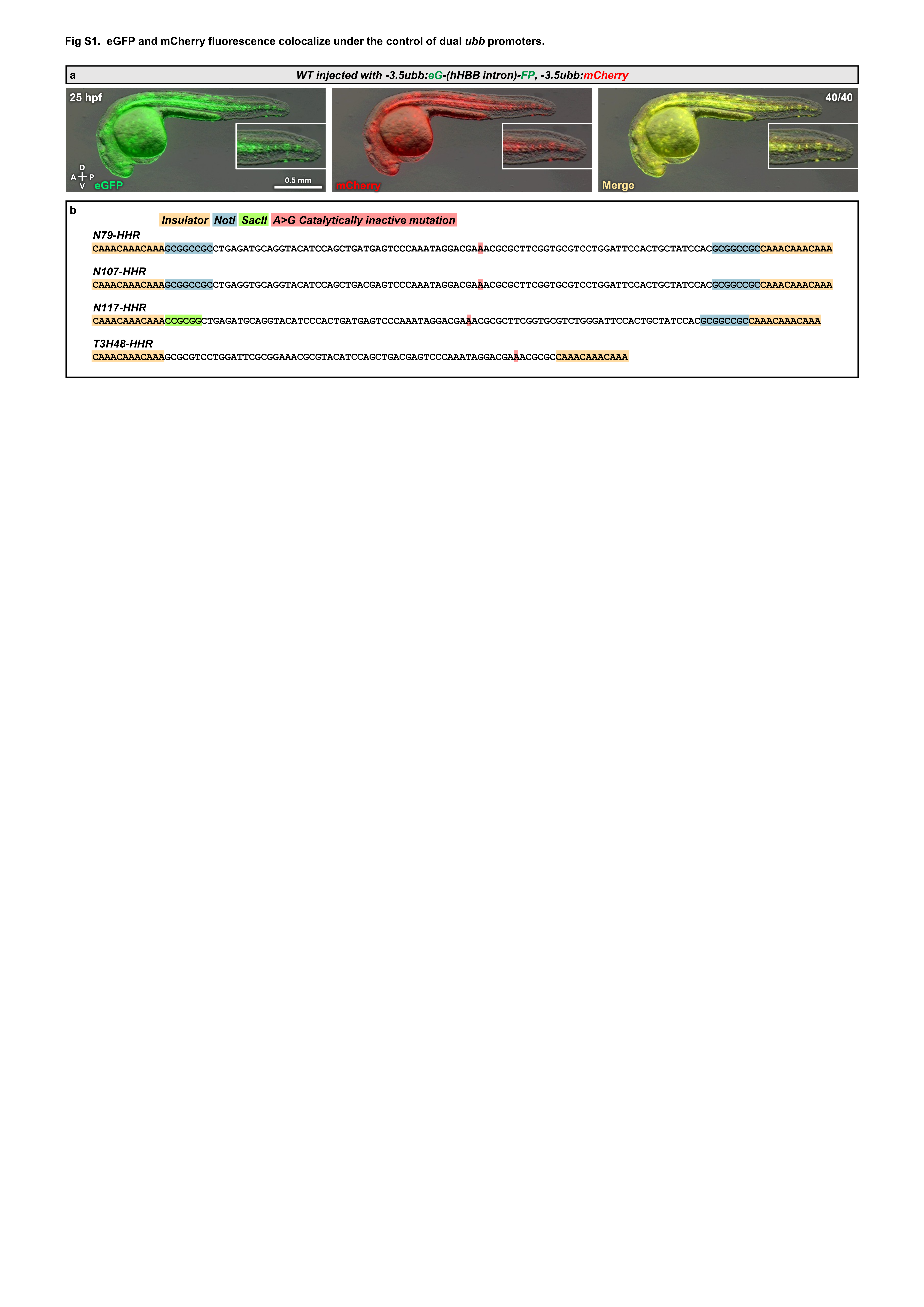

Supplement: S1 Fig — (a) Merge of brightfield and fluorescence images of 25 hpf embryos injected at the one-cell stage with the dual ubb vector; the proportion of embryos matching the image shown is indicated in the top right corner. (b) Sequences of the N79, N107, N107, and T3H48 hammerhead ribozymes used in this study and of the flanking insulators, and location of the inactivating mutation. (TIF) [file pgen.1011594.s001.tif]

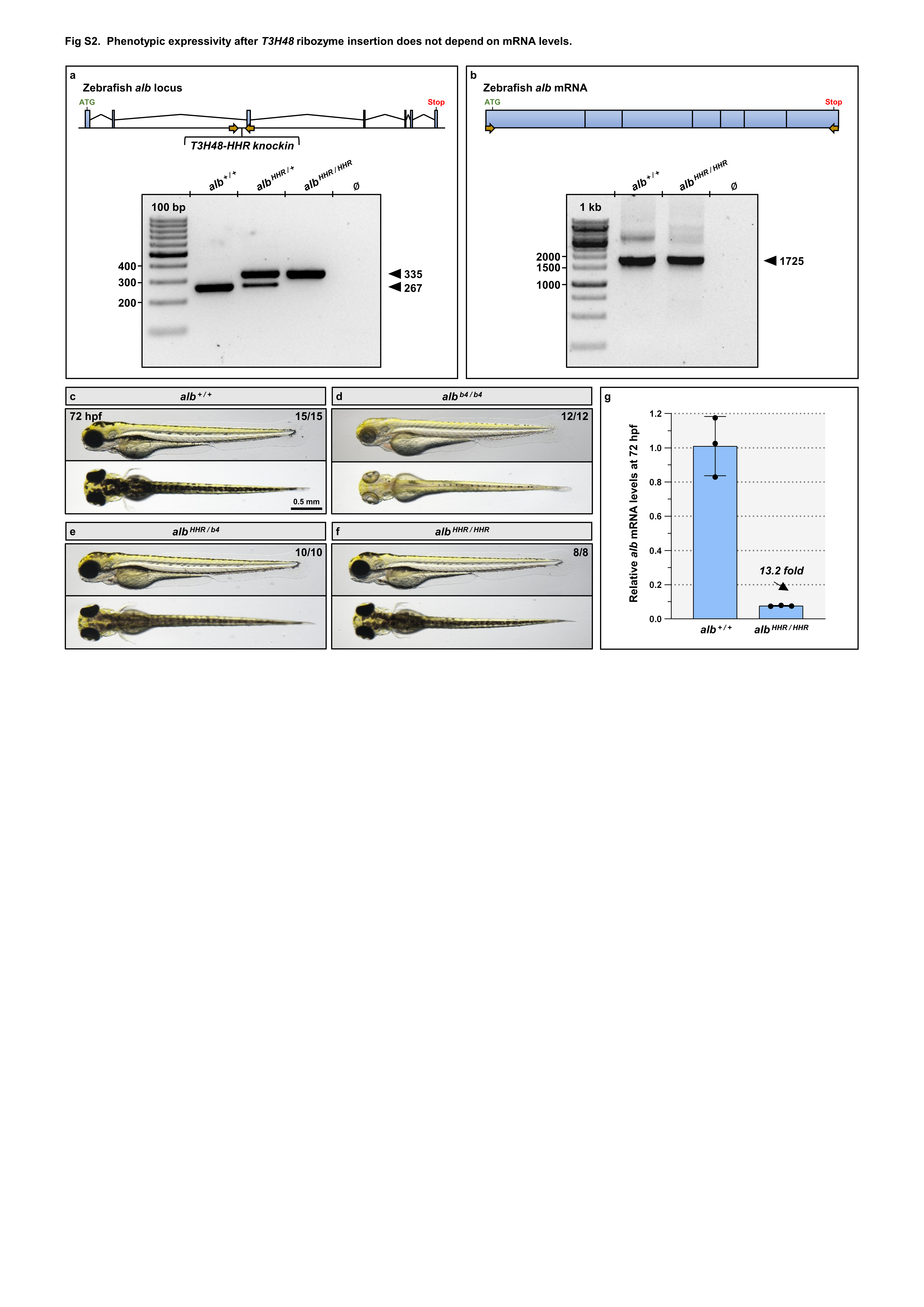

Supplement: S2 Fig — (a) Schematic of the alb locus and agarose gel image showing a PCR amplification of the albHHR region from 36 hpf wild-type, albHHR/+, and albHHR/HHR embryos. (b) Schematic of the alb mRNA and agarose gel image showing an RT-PCR amplification of the alb full-length mRNA from 36 hpf wild-type and albHHR/HHR embryos. (c-f) Brightfield images of 72 hpf wild-type (c), albb4/b4 (d), albHHR/b4 (e), and albHHR/HHR (f) larvae. (g) Relative alb mRNA levels in 72 hpf wild-type and albHHR/HHR larvae; n=3 biologically independent samples; Ct values are listed in S1 Table. The proportion of larvae matching the image shown is indicated in the top right corner of each image. (TIF) [file pgen.1011594.s002.tif]

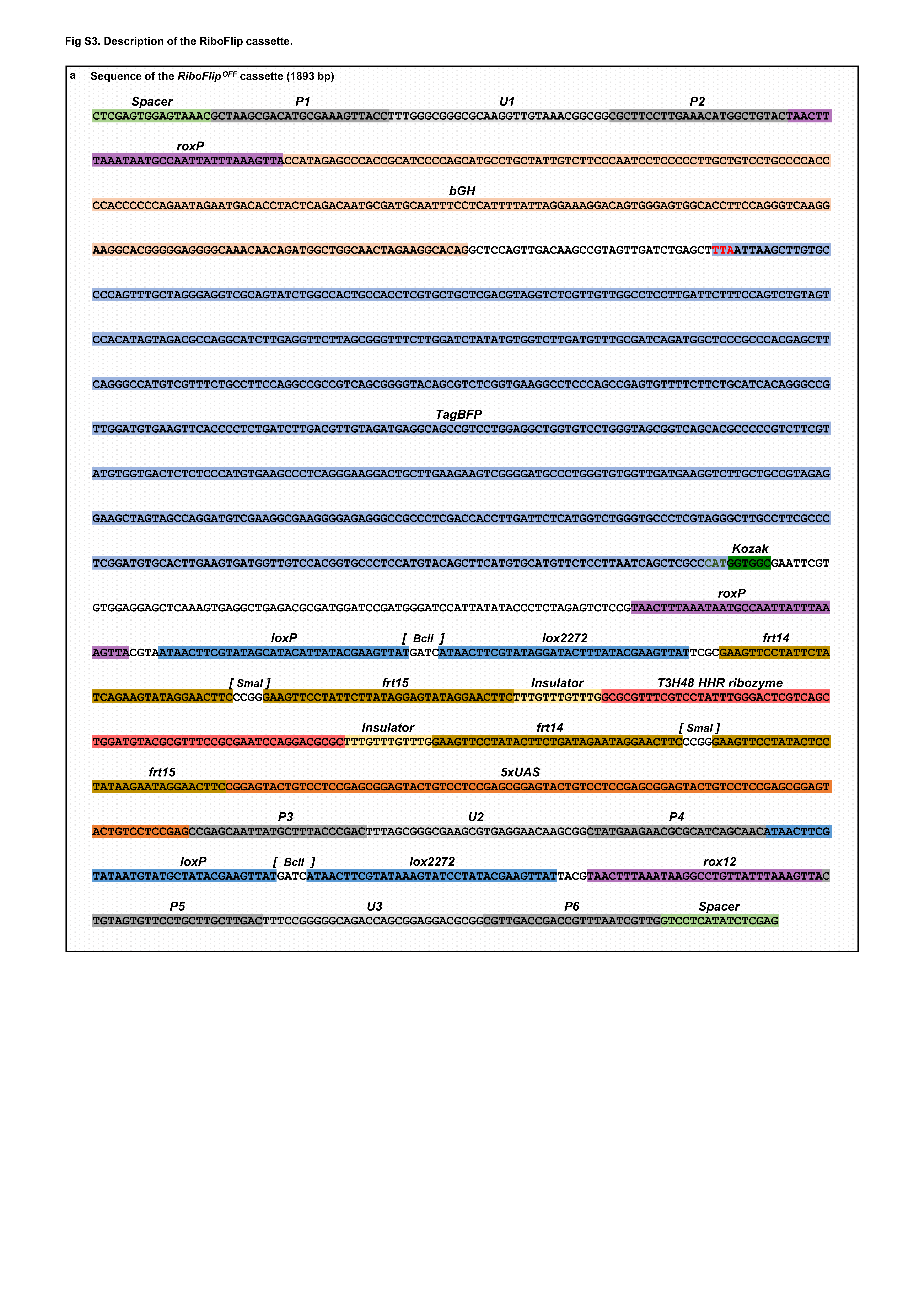

Supplement: S3 Fig — (a) Annotated sequence of the RiboFlip cassette containing an extensive list of all components, including six unique primer sites (P1-6), three universal CRISPR sites (U1-3), a β-globin terminator (bGH) downstream of the TagBFP, and recombination sites (LOX/FRT/ROX). (TIF) [file pgen.1011594.s003.tif]

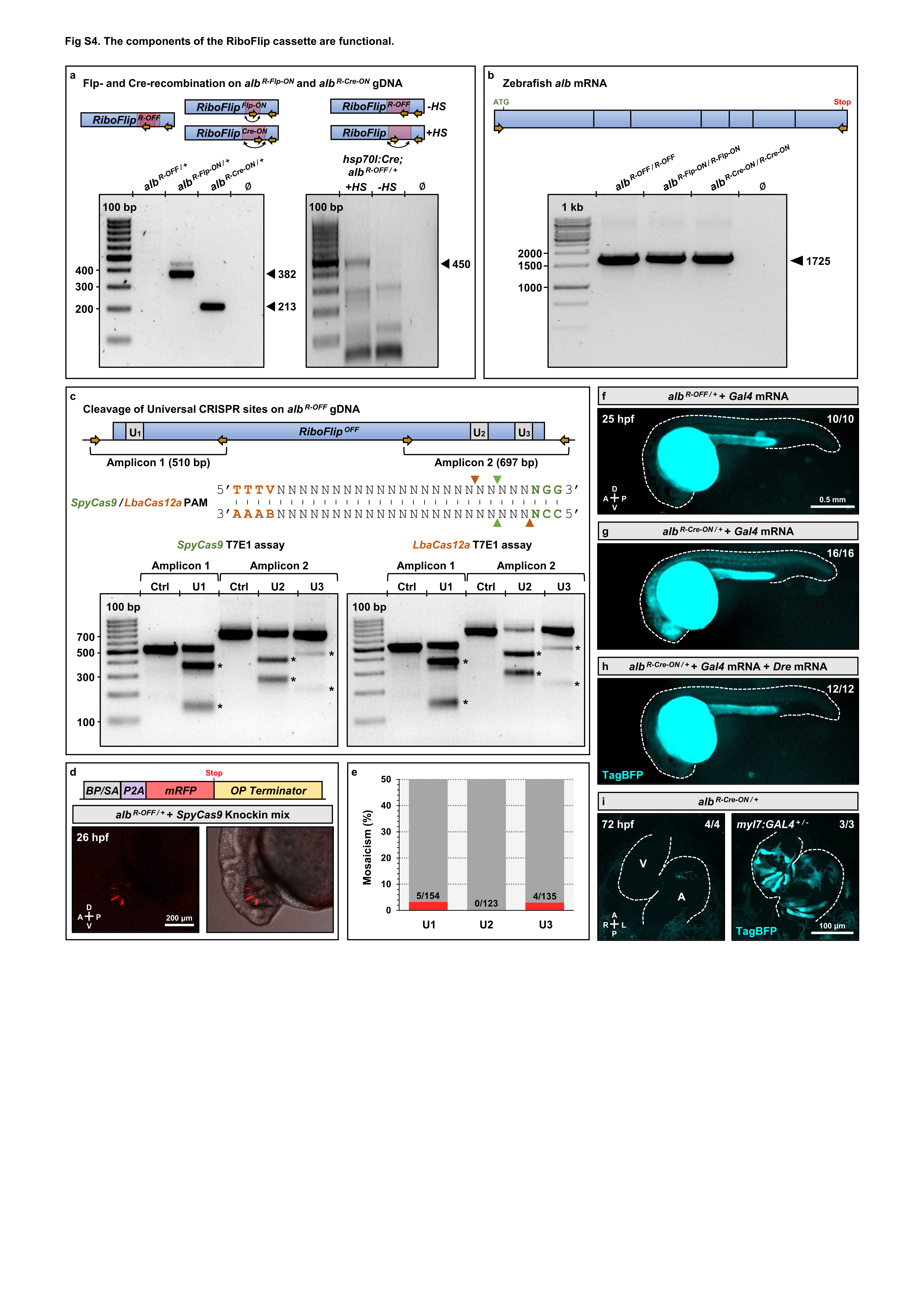

Supplement: S4 Fig — (a) Schematics and agarose gel images of the RiboFlip cassette inserted in the alb locus showing a Flp and Cre flipping-specific PCR amplification of the indicated fragments from 36 hpf albR-OFF/+, albR-Flp-ON/+, and albR-Cre-ON/+ embryos (left panel), and from albR-OFF/+; hsp70l:Cre embryos without or with heat shock (HS) (right panel); the purple boxes represent the portion of the RiboFlip cassette that gets flipped upon Flp- or Cre-induction; heat shock was performed by placing 24 hpf embryos in pre-heated egg water at 39°C for 1 h three times in a row, spaced by 1 h at 28°C; two different primer pairs are used to visualize Cre mRNA- and hsp70l:Cre-mediated recombination. (b) Schematic and agarose gel image of the alb mRNA showing a RT-PCR amplification of alb full-length mRNA from 36 hpf albR-OFF/R-OFF, albR-Flp-ON/R-Flp-ON, and albR-Cre-ON/R-Cre-ON embryos. (c) Schematic and sequence of the RiboFlip cassette inserted in the alb gene and the universal SpyCas9/LbaCas12 CRISPR sites (U-CRISPR), and agarose gel image of the T7 endonuclease I (T7EI) assay performed on 48 hpf albR-OFF/R-OFF embryos; asterisks (*) highlight the degradation products of the T7EI assay. (d) Donor cassette schematic and fluorescence image of a 26 hpf albR-OFF/+ embryo injected at the one-cell stage with Cas9 protein, a U-CRISPR-targeting sgRNA, and a 5’AmC6-modified donor PCR product consisting of a branch point/splice acceptor (BP/SA) sequence, a P2A peptide, a mRFP reporter, and an ocean pout (OP) terminator (U-CRISPR mix). (e) Proportion of positive embryos expressing mRFP in retinal pigmented epithelial cells following injection with U1, U2, and U3 SpyCas9 U-CRISPR mix. (f-h) Fluorescence images of 25 hpf albR-OFF (f) and albR-Cre-ON/+ (g,h) embryos injected at the one-cell stage with Gal4 (f,g) or Gal4 and Dre (h) mRNA. (i) Confocal images of hearts (ventral views) from 72 hpf albR-Cre-ON/+ and albR-Cre-ON/+; myl7:GAL4+/- larvae; maximum z-projection; annotations correspond to the he [file pgen.1011594.s004.tif]
