# Supplementary material for: A recombinase-activated ribozyme to knock down endogenous gene expression in zebrafish
Source: PLoS Genet. 2025 Feb 7;21(2):e1011594. doi: 10.1371/journal.pgen.1011594 (PMC11856399; doi:10.1371/journal.pgen.1011594)
Supplement: S1 Table — (PDF) [file pgen.1011594.s005.pdf]

Supplementary table S1. CRISPR site, donors, primers, and Ct values.

CRISPR sites (5'-3', **PAM**)	
Knockins in the second intron of the *alb* locus	GATGTAAATGACTGCAACTG**AGG**
Base editing of *T3H48-HHR* inserted in the *alb* locus	ACGAAACGCGCCAACAACTG**AGG**

Donor ssODN (5'-3', homology arms in lowercase)	
ssODN to insert the *T3H48-HHR*	gagtgatgtaaatgactgcaaCAACAAGCGCGTCCTGGATTCGCGGAAACGCGTACA

Donor PCR products (5'AmC6 modified, homology arms in lowercase)	Primer 1 (5'-3')	Primer 2 (5'-3')
Knockin of the RiboFlip	aacattttccaccaaacccttgattctg	aaattcctcctcaagaaatcaaaagtct
Knockin of the mRFP reporter in the U-CRISPR 1 of the RiboFlip	gctaagcgacatgcgaaagttacctttg	tggcattatttaaagttagtacagccat
Knockin of the mRFP reporter in the U-CRISPR 2 of the RiboFlip	ccgagcaattatgctttacccgacttta	tacattatacgaagttatgttgctgatg
Knockin of the mRFP reporter in the U-CRISPR 3 of the RiboFlip	ctgtagtgttcctgcttgcttgactttc	agctcgagatatgaggaccaacgattaa

PCR genotyping on gDNA	Primer 1 (5'-3')	Primer 2 (5'-3')
alb^{HHR} and *alb*^{wt} (Fig. S2a)	TGCAGTCAAAATCATCCATCG	TACTACCAGGCATTCTGCTCAC
alb$^{R\text{-}OFF}$	TGCAGTCAAAATCATCCATCG	GTACAGCCATGTTTCAAGGAAGCG
alb$^{R\text{-}Flp\text{-}ON}$ and *alb*$^{R\text{-}Cre\text{-}ON}$ (Fig. S4a left panel)	GCTGACGAGTCCCAAATAGG	GTCAAGCAAGCAGGAACACTACAG
alb$^{R\text{-}Cre\text{-}ON}$ with *hsp70l:Cre* (Fig. S4a, right panel)	GTCGGGTAAAGCATAATTGCTCGG	GTCAAGCAAGCAGGAACACTACAG
T7E1 Amplicon 1 *alb*$^{R\text{-}OFF}$ (Fig. S4c)	TGCAGTCAAAATCATCCATCG	ATACTGCGACCTCCCTAGCAAAC
T7E1 Amplicon 2 *alb*$^{R\text{-}OFF}$ (Fig. S4c)	CCTATTTGGGACTCGTCAGC	TACTACCAGGCATTCTGCTCAC
Dre recombination on *alb*$^{R\text{-}Cre\text{-}ON}$ (Fig. S4f-h)	GCTAAGCGACATGCGAAAGTTACC	CAACGATTAAACGGTCGGTCAACG
Gal4 specific (Fig. S4i)	GCTACTGTCTTCTATCGAACAAGC	GTTACCCGGGAGCATATCGAGATC

PCR amplification on cDNA	Primer 1 (5'-3')	Primer 2 (5'-3')
alb full length cDNA amplification (Fig. S2b, S4b)	CTCAACTATGCACTAGGAATCAAG	GAGGCTTATTCTGTACATC

RT-qPCR amplification on cDNA	Primer 1 (5'-3')	Primer 2 (5'-3')
mCherry	GCCAAGCTGAAGGTGACCAAG	CCGTCCTCGAAGTTCATCAC
mature *split-GFP*	AAGTCCGCCATGCCCGAAGGGTAC	TGCTCAGGTAGTGGTTGTCG
eef1b2	ATCTGTTTGGCTCCGATGAG	CAGGCTTCTTTGCCTTCTTG
mature *alb*	CGGACCCATTAAAGCCTATTTG	GGCTCTTTCATCAGTGGCTTCTC

RT-qPCR target	Sample	Ct value
mCherry (Fig. 1b)	inactive N79 (1)	21,63693129
mCherry (Fig. 1b)	inactive N79 (2)	21,69801297
mCherry (Fig. 1b)	inactive N79 (3)	21,72693123
mCherry (Fig. 1b)	inactive N107 (1)	23,44735992
mCherry (Fig. 1b)	inactive N107 (2)	23,41967644
mCherry (Fig. 1b)	inactive N107 (3)	23,42454409
mCherry (Fig. 1b)	inactive N117 (1)	25,13418539
mCherry (Fig. 1b)	inactive N117 (2)	25,00288846
mCherry (Fig. 1b)	inactive N117 (3)	25,02536596
mCherry (Fig. 1b)	inactive T3H48 (1)	21,74914615
mCherry (Fig. 1b)	inactive T3H48 (2)	21,58547245
mCherry (Fig. 1b)	inactive T3H48 (3)	21,80082062
mCherry (Fig. 1b)	inactive 3xT3H48 (1)	22,85511803
mCherry (Fig. 1b)	inactive 3xT3H48 (2)	23,08365768
mCherry (Fig. 1b)	inactive 3xT3H48 (3)	23,11806774
mCherry (Fig. 1b)	active N79 (1)	23,35025323
mCherry (Fig. 1b)	active N79 (2)	23,47819573
mCherry (Fig. 1b)	active N79 (3)	25,09848533
mCherry (Fig. 1b)	active N107 (1)	22,50012619
mCherry (Fig. 1b)	active N107 (2)	22,51271175
mCherry (Fig. 1b)	active N107 (3)	22,47587895
mCherry (Fig. 1b)	active N117 (1)	22,61420872
mCherry (Fig. 1b)	active N117 (2)	22,51198514
mCherry (Fig. 1b)	active N117 (3)	22,58198401
mCherry (Fig. 1b)	active T3H48 (1)	22,47499334
mCherry (Fig. 1b)	active T3H48 (2)	22,34064806
mCherry (Fig. 1b)	active T3H48 (3)	22,34527808
mCherry (Fig. 1b)	active 3xT3H48 (1)	23,40827441
mCherry (Fig. 1b)	active 3xT3H48 (2)	23,32338042
mCherry (Fig. 1b)	active 3xT3H48 (3)	23,45790727
mature split-GFP (Fig. 1b)	inactive N79 (1)	20,85906268
mature split-GFP (Fig. 1b)	inactive N79 (2)	20,82843349
mature split-GFP (Fig. 1b)	inactive N79 (3)	20,64558375
mature split-GFP (Fig. 1b)	inactive N107 (1)	23,48594153
mature split-GFP (Fig. 1b)	inactive N107 (2)	23,52172806
mature split-GFP (Fig. 1b)	inactive N107 (3)	23,34349879
mature split-GFP (Fig. 1b)	inactive N117 (1)	25,11737879

mature split-GFP (Fig. 1b)	inactive N117 (2)	25,0689156
mature split-GFP (Fig. 1b)	inactive N117 (3)	25,20605878
mature split-GFP (Fig. 1b)	inactive T3H48 (1)	20,20023368
mature split-GFP (Fig. 1b)	inactive T3H48 (2)	20,356278
mature split-GFP (Fig. 1b)	inactive T3H48 (3)	20,43548158
mature split-GFP (Fig. 1b)	inactive 3xT3H48 (1)	22,88987943
mature split-GFP (Fig. 1b)	inactive 3xT3H48 (2)	22,87757882
mature split-GFP (Fig. 1b)	inactive 3xT3H48 (3)	23,03493086
mature split-GFP (Fig. 1b)	active N79 (1)	23,61964605
mature split-GFP (Fig. 1b)	active N79 (2)	23,81316574
mature split-GFP (Fig. 1b)	active N79 (3)	23,85464462
mature split-GFP (Fig. 1b)	active N107 (1)	22,17642027
mature split-GFP (Fig. 1b)	active N107 (2)	21,82938805
mature split-GFP (Fig. 1b)	active N107 (3)	21,53816165
mature split-GFP (Fig. 1b)	active N117 (1)	23,41803475
mature split-GFP (Fig. 1b)	active N117 (2)	23,43053082
mature split-GFP (Fig. 1b)	active N117 (3)	23,30900872
mature split-GFP (Fig. 1b)	active T3H48 (1)	25,32208645
mature split-GFP (Fig. 1b)	active T3H48 (2)	25,49943467
mature split-GFP (Fig. 1b)	active T3H48 (3)	25,33089349
mature split-GFP (Fig. 1b)	active 3xT3H48 (1)	27,13019776
mature split-GFP (Fig. 1b)	active 3xT3H48 (2)	26,96258712
mature split-GFP (Fig. 1b)	active 3xT3H48 (3)	26,94612472

eef1b2 (Fig. 2b)	$alb^{+/+}$ (1)	18,89674315
eef1b2 (Fig. 2b)	$alb^{+/+}$ (2)	18,71227693
eef1b2 (Fig. 2b)	$alb^{+/+}$ (3)	18,61422978
eef1b2 (Fig. 2b)	$alb^{HHR/HHR}$ (1)	18,45665081
eef1b2 (Fig. 2b)	$alb^{HHR/HHR}$ (2)	18,27543416
eef1b2 (Fig. 2b)	$alb^{HHR/HHR}$ (3)	18,19189318
mature alb (Fig. 2b)	$alb^{+/+}$ (1)	23,95448715
mature alb (Fig. 2b)	$alb^{+/+}$ (2)	23,84783465
mature alb (Fig. 2b)	$alb^{+/+}$ (3)	23,75252819
mature alb (Fig. 2b)	$alb^{HHR/HHR}$ (1)	27,07370947
mature alb (Fig. 2b)	$alb^{HHR/HHR}$ (2)	27,36288004
mature alb (Fig. 2b)	$alb^{HHR/HHR}$ (3)	27,15270079

eef1b2 (Fig. S2g)	$alb^{+/+}$ (1)	17,07876327
eef1b2 (Fig. S2g)	$alb^{+/+}$ (2)	17,02011258
eef1b2 (Fig. S2g)	$alb^{+/+}$ (3)	17,49278021
eef1b2 (Fig. S2g)	$alb^{HHR/HHR}$ (1)	17,11790448
eef1b2 (Fig. S2g)	$alb^{HHR/HHR}$ (2)	17,0921248
eef1b2 (Fig. S2g)	$alb^{HHR/HHR}$ (3)	17,17129248
mature alb (Fig. S2g)	$alb^{+/+}$ (1)	22,78111652
mature alb (Fig. S2g)	$alb^{+/+}$ (2)	22,41701141
mature alb (Fig. S2g)	$alb^{+/+}$ (3)	22,69304632
mature alb (Fig. S2g)	$alb^{HHR/HHR}$ (1)	26,28471419
mature alb (Fig. S2g)	$alb^{HHR/HHR}$ (2)	26,2687825
mature alb (Fig. S2g)	$alb^{HHR/HHR}$ (3)	26,25541975

eef1b2 (Fig. 3b)	$alb^{R\text{-}OFF/R\text{-}OFF}$ (1)	21,13498785
eef1b2 (Fig. 3b)	$alb^{R\text{-}OFF/R\text{-}OFF}$ (2)	21,25677862
eef1b2 (Fig. 3b)	$alb^{R\text{-}OFF/R\text{-}OFF}$ (3)	21,07964803
eef1b2 (Fig. 3b)	$alb^{R\text{-}Flp\text{-}ON/R\text{-}Flp\text{-}ON}$ (1)	20,99242207
eef1b2 (Fig. 3b)	$alb^{R\text{-}Flp\text{-}ON/R\text{-}Flp\text{-}ON}$ (2)	20,81960316
eef1b2 (Fig. 3b)	$alb^{R\text{-}Flp\text{-}ON/R\text{-}Flp\text{-}ON}$ (3)	20,85895457
eef1b2 (Fig. 3b)	$alb^{R\text{-}Cre\text{-}ON/R\text{-}Cre\text{-}ON}$ (1)	21,08644761
eef1b2 (Fig. 3b)	$alb^{R\text{-}Cre\text{-}ON/R\text{-}Cre\text{-}ON}$ (2)	20,94223588
eef1b2 (Fig. 3b)	$alb^{R\text{-}Cre\text{-}ON/R\text{-}Cre\text{-}ON}$ (3)	21,01064484
mature alb (Fig. 3b)	$alb^{+/+}$ (1)	24,93940702
mature alb (Fig. 3b)	$alb^{+/+}$ (2)	24,85969632
mature alb (Fig. 3b)	$alb^{+/+}$ (3)	24,94715454
mature alb (Fig. 3b)	$alb^{R\text{-}Flp\text{-}ON/R\text{-}Flp\text{-}ON}$ (1)	28,57857809
mature alb (Fig. 3b)	$alb^{R\text{-}Flp\text{-}ON/R\text{-}Flp\text{-}ON}$ (2)	28,26325748
mature alb (Fig. 3b)	$alb^{R\text{-}Flp\text{-}ON/R\text{-}Flp\text{-}ON}$ (3)	28,61662514
mature alb (Fig. 3b)	$alb^{R\text{-}Cre\text{-}ON/R\text{-}Cre\text{-}ON}$ (1)	28,00190743
mature alb (Fig. 3b)	$alb^{R\text{-}Cre\text{-}ON/R\text{-}Cre\text{-}ON}$ (2)	27,7961582
mature alb (Fig. 3b)	$alb^{R\text{-}Cre\text{-}ON/R\text{-}Cre\text{-}ON}$ (3)	27,97793081
