# Supplementary material for: A recombinase-activated ribozyme to knock down endogenous gene expression in zebrafish
Source: PLoS Genet. 2025 Feb 7;21(2):e1011594. doi: 10.1371/journal.pgen.1011594 (PMC11856399; doi:10.1371/journal.pgen.1011594)
Supplement: Attachment — Submitted filename: Rebuttal_tjuan.pdf [file pgen.1011594.s007.pdf]

Max-Planck-Institute
for Heart and Lung Research
W.G. Kerckhoff-Institute

Jeffrey J Essner
Guest Editor
PLOS Genetics

22 December 2024

Aleksandra Trifunovic
Section Editor
PLOS Genetics

Dear Aleksandra, dear Jeff,

Please find attached our revised manuscript entitled "**A recombinase-activated ribozyme to knock down endogenous gene expression in zebrafish**" for consideration as an article in *PLOS Genetics*. We thank you for your initial positive response, which has given us the opportunity to improve our manuscript.

We also want to thank the reviewers for their positive remarks as well as their helpful suggestions and advice. Please find below our point-by-point response to the reviewers' comments.

We have now addressed all of the major and minor comments from the reviewers. In particular, we have used the state-of-the-art ABE-ultramax base editing system (Qin et al., 2024) to inactivate the ribozyme in the endogenous *albino* gene, as an additional control for our experiments. We also show that the RiboFlip element can be used as a landing site for additional sequences using CRISPR/Cas9. In addition, we have softened and clarified the language in the manuscript according to the reviewers' suggestions. Overall, our data indicate that self-cleaving ribozymes offer a new approach to control endogenous gene expression in vertebrates and introduce RiboFlip to control ribozyme cleavage using recombinases.

All major changes in the main text are highlighted in yellow.

Thank you for considering our revised manuscript.

With best regards,

Thomas Juan and Didier Stainier

Reviewer #1 comments:

The manuscript by Juan et al. describes the use of a ribozyme to knockdown endogenous gene expression in zebrafish. The strategy is elegant in that the ribozyme sequence is integrated into the intron of using CRISPR/Cas9 gene editing so that the preRNA is degraded following transcription. Several different ribozymes were tested for this purpose to determine the most efficient system. This represents the first time such a system has been used in vertebrates and has the potential to be a useful genetic tool. The efficiency of the system has the potential to induce phenotypic changes and displays approximately 13-fold knockdown of the golden transcript. However, to make the system of broad utility greater knockdown would be needed, as even at the golden locus, the phenotype recovers over time. This should not detract the achievement from the applying this for the first time in vertebrates, but it is highly recommended that the language is softened and discussion of the degree of knockdown for efficient application is discussed. For example, as little as 5% of activity for many genes is sufficient to not produce a phenotype.

The system is then used with recombinase systems to flip the ribozyme so that it is no longer expressed on the RNA level in the correct orientation. This inactivates the ribozyme and allows the system to be used for conditional genetics. This is a step forward for conditional genetics, but the incomplete knockdown may make phenotypic analysis complicated. Nevertheless, this would be the end goal of integrating the ribozyme into an intron. Overall, the manuscript represents an important step forward for using ribozymes for conditional genetics and a platform to improve knockdown to the point where the system would have widespread use.

We thank the reviewer for their supportive comments.

Please address the following minor points prior to publication.

Soften the language for this being an efficient system throughout the manuscript.

We have softened the language throughout the manuscript regarding the efficiency of the system.

Throughout the results, the conclusions of the experiment are often stated without describing the rationale or the result itself leaving the reader to try to fill in the blanks. For example, it is simply stated that "that the reduction occurs independently of splicing defects." This is just one example of many instances where the results section could be expanded to help the reader. It also recommended the strategy for using different loxp and frt sites be explained. Having outcomes/predictions following recombinase delivery (especially because this is different for different recombinases) will be important for most readers.

We have now clarified the rationale for the experiments in the Results (lines 156-161, 163, 182-184, 203-207) and the Methods (lines 376-377) sections.

Please define all abbreviations used in the figures in the figure legends.

We have defined all abbreviations used in the figures in the figure legends.

Reviewer #2 comments:

Juan et al. examined the utility of T3H48 ribozyme for conditional gene knockdown in zebrafish. The authors demonstrate that this ribozyme can inactivate the expression of an endogenous gene when inserted in an intron of the target gene. They created an invertible cassette called RiboFlip, which does not suppress gene expression when inserted in the reverse orientation but does so only after the Cre- or Flp-mediated cassette inversion. The genetic approach for the conditional inactivation of gene expression is still limited in zebrafish, and the approach described in this study will be a valuable addition to the genetic toolbox available in the community. I have several comments and questions about the study, as described below:

We thank the reviewer for their supportive comments.

1) I like the study, but I am a bit concerned about the lack of a convincing example of a loss-of-function phenotype achieved with the presented method. The pigments eventually return in alb-HHR/HHR (Fig. S2e, f). Is such a phenotype typical for the loss-of-function experiment with the alb gene?

We have now added comments in the discussion regarding the expressivity of the described phenotype (lines 265-271).

2) I am confused with Fig. S2b. How could alb-HHR/HHR embryos show fewer pigments at 36 hpf (Fig. 2f), although they seem to have mature alb-mRNA? (Fig. S2b).

The RT-PCR we show in Fig. S2b was at saturation, to show that the *alb* mRNA produced in *alb*$^{HHR/HHR}$ embryos is of wild-type size. This experiment is intended to test whether ribozyme cleavage, and not the perturbation of pre-mRNA splicing, is responsible for the pigmentation phenotype. We have now expanded the description of the experiment in the Results section (lines 182-184). In addition, we have now inactivated the ribozyme in the *alb* gene using the ABE-ultramax base editing method (Qin et al., 2024) to further test whether ribozyme cleavage, rather than splicing defects, is responsible for the pigmentation phenotype (Fig. 2g-k).

3) The purpose of using the insulator may need more explanation. It would also be appreciated if the authors could provide more details about the insulator used in this study, as it is not the one commonly used in transgenic constructs.

We have now added details about the insulator in the Results (lines 156-158, 163, 178-180) and Methods (lines 376-377) sections.

4) Does this method degrade mRNA through NMD and cause genetic compensation?

We agree with the reviewer about the importance of this question and are currently preparing a separate manuscript using an established genetic compensation model to address it.

5) Fig. 3a, RiboFlip-Cre-ON panel: It might be better to remove the shade over TagBFP to make it clearer that Cre-mediated recombination induces TagBFP expression under the control of the UAS promoter.

Noted and done.

6) Please clarify the labeling in Fig. 3b: R-OFF-/-, R-Flp-ON-/-, and R-Cre-ON-/-.

Noted and done.

7) It would be better to include positive controls in Fig. S4a.

We have now repeated the PCR by including an additional primer pair external to the RiboFlip cassette, that amplifies a band of 316 bp, visible between the 213 bp of the *Cre* amplicon, and the 382 bp of the *Flp* amplicon:

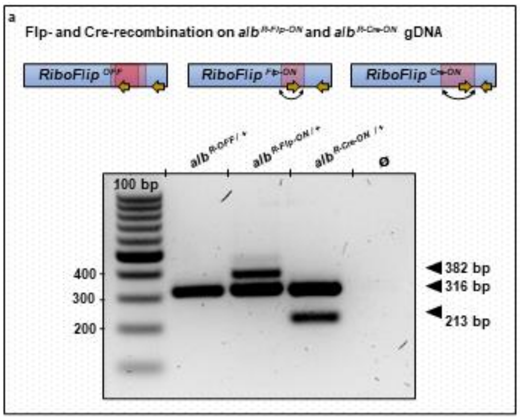

Schematics and agarose gel image of the RiboFlip cassette inserted in the *alb* locus showing a Flp and Cre flipping-specific PCR amplification of the indicated fragments from 36 hpf $alb^{R\text{-}OFF/+}$, $alb^{R\text{-}Flp\text{-}ON/+}$, and $alb^{R\text{-}Cre\text{-}ON/+}$ embryos; the purple boxes represent the part of the RiboFlip cassette that gets flipped upon Flp- or Cre-induction.

We have now also shown that an *hsp70l:Cre* transgene can induce RiboFlip recombination after heat shock (Fig. S4a). However, in this experiment, the amplification of the positive control outcompetes the amplification of the recombined sequence because the recombination is not complete. In order to be consistent, we decided to retain the previous gel image.

8) It would be helpful if major base pair sizes were indicated with the DNA ladders.

Noted and done.

Reviewer #3 comments:

The manuscript of Juan et al. describes the use of a ribozyme to perform gene Knock-Down (KD) in Zebrafish, a vertebrate model.
The manuscript is direct and clear, and the Zebrafish and other vertebrate communities can benefit to the implementation of this new molecular tool for advanced genetic manipulations. Although I appreciate that the manuscript is direct, it could be really strengthened by providing more data and applications

We thank the reviewer for their supportive comments.

Main comments:
-While the characterization of various ribozymes on a dual coding transgene (eGFP/mCherry) is a great starting point, in vivo applications are limited to the albino gene. It seems that the manuscript will be stronger and could lead to a wide adoption of the ribozyme if the authors use their system on other genes, and not be limited to such a 'generic gene'. Describing, even superficially, some new biological functions by KD several endogenous genes will be beneficial and will increase the impact of this study.

We appreciate the reviewer's suggestion to explore additional targets. While we agree that applying the RiboFlip system to other genes would strengthen the study, generating new knockin lines is beyond the scope of this initial manuscript.

-The authors propose ribozyme-KD as a way to extend the set of tools for genetic manipulation in Zebrafish and possibly in other vertebrate models. Therefore, it would be interesting to describe other tools available in Zebrafish and vertebrate in general (eg RNAi, shRNA) and describe why a ribozyme could be a unique tool for gene KD.

We have now added comments in the discussion to compare ribozyme KD with other RNA targeting tools (lines 301-306).

-Since one of the usefulness of a ribozyme is to KD genes, the authors should use it to KD a gene for which KO allele cannot be obtained because mutants are not viable.

We have now added comments in the discussion to mention that ribozyme KD can be a great asset when KO results in lethal phenotypes (lines 271-272).

-The authors state that their system is conditional. While true, injecting Flp or Cre is still cumbersome. It would be interesting to have these enzymes under the control of a heat-shock promoter to make the tool versatile and easy to use for the community. Also, I am not sure of what the authors mean by the advantage of "the visualization of Cre recombined cells independently of the targeted gene expression", with the example of the cardiomyocyte regulatory element. This is because always 100% of the Cre injected embryos are not mosaic for recombination? Because having the recombination marker active in the same cells that express the gene targeted could be practical, since it would show that for sure the ribozyme is active in these cells. Moreover, having mosaic recombination with same cell marker can be better, as commonly done in Drosophila and Flp/ Heat-Shock for cell autonomous effect.

We have now added data showing that an *hsp70l:Cre* transgene can induce recombination in $alb^{R\text{-}OFF/+}$ animals, similar to *Cre* mRNA injection (Fig. S4a). Regarding the cardiomyocyte regulatory element, we wanted to emphasize that recombination is visible in our system even when *alb* is not expressed. When a recombination reporter is under the control of the target gene, recombination cannot be visualized before gene expression begins or after it ends, which can limit applications such as cell lineage tracing. We have now added more details on the rationale for this experiment (lines 220-223).

-The authors could try, instead of three ribozyme copies in the same intron (which is less efficient than a single ribozyme copy), to put several ribozyme copies spread on different introns. If such strategy improves the KD, this could really justify using ribozymes over other KD strategies… For instance, the authors could study a gene with a dose response phenotype.

While the generation of another knockin line is beyond the scope of this manuscript, we agree with the reviewer that putting several ribozyme copies spread over multiple introns is a good idea. We have now also mentioned in the discussion that UTR insertions should be evaluated since they would induce pre-mRNA and mRNA cleavage (lines 251-252).

Minor comments:
-The sentence "eGFP split by a human B-globin intron" might be confusing in regard of the split GFP system. Maybe something like "eGFP coding sequence containing an intron" could be use…

We have revised the sentence accordingly.

-Only in the discussion the recovering of pigmentation is explained, this should be done in the result part. How the authors rule out loss of ribozyme activity overtime or during some developmental stage?

We have added further clarification in the Results section (lines 182-184) and show that *alb* levels are still strongly reduced even as pigmentation recovers (Fig. S2g).

-The discussion seems redundant with other manuscript part, with sometime very similar sentences.

We have reduced redundancies.

-Insulators are mentioned in material and methods and figures, but not explained in the main text.

We have now added details regarding the insulators in the main text (lines 156-158, 163, 178-180).

-Does the insulator and ribozyme insertion could affect expression of the targeted gene and neighbor
genes? Use of the inactive ribozyme and quantitative PCR or other methods could be performed.

To investigate these questions, we used the recently published ABE-ultramax base editing system (Qin et al., 2024) to convert the active ribozyme into an inactive one (Fig. 2g-k). This conversion rescued the pigmentation phenotype in embryos, indicating that it is the cleavage of the ribozyme and not the insertion itself that is responsible for this phenotype (lines 189-193).

-Fig S2b legend: RT-PCR and not just PCR amplification?

We have now corrected this mistake.

-Not sure if calling the mCherry transgene a housekeeping gene is correct. It is more a control or reference gene.
-The Tol2 transgene is a verified single copy?

We now refer to *mCherry* as a reference gene throughout the manuscript. The Tol2 experiment is analyzed in F0 embryos, where multiple copies of the injected plasmid can be present, making the use of the *mCherry* essential to normalize the number of copies. We have now added more information regarding this experiment (lines 158-161).

-Fig1a: the 3xSV40 utr in 3 yellow box is confusing since it looks like there are small introns inside. Why using 3 copies?

We have now removed the spaces between the *SV40 UTRs* (Fig. 1a), and added details in the main text to explain that the 3 copies are used to prevent transcription readthrough and the inclusion of *mCherry* in the *eGFP* transcript (line 149).

-Fig1a: a schematic of how ribozyme works could be very helpful for readers.

We have now added some details of how the ribozyme works, including the secondary structure of the ribozyme and its cleavage products (Fig. 1a)

-Fig2a: the fact that the exogenous CRISPR sites in the sequence part are in light blue, as the exons in the gene model, is confusing.

We have now changed the color code for the CRISPR sites (Fig. 2) to match that of the universal CRISPR sites in the RiboFlip cassette (Fig. S3).

-Ribozyme testing in homozygous lines should be better explained. How the crosses have been made, from homozygous parents, or Het providing wildtype maternal mRNA?

We have now added details of the crosses in the methods section (lines 354-356), including evidence that *alb* mRNA is not maternally provided.

-For non-zebrafish readers, the genotype can be not clear in Fig4. It looks like it is for both background one allele with RiboFlip active (R-Flp-On or R-Cre-ON), and the other allele corresponds to two different mutant alleles (bns732 and bns733). Some precision in the legend could be useful for a broader readership.

We have now simplified the genotype and the legend of this figure.

-Some explanation about the difference between roxP-12, frt14/15, and loxP/2272 in Fig3a could also be useful

We have now added information about the different recombinase sites (lines 203-207, 295-298, 386-388).

-Introducing exogenous/universal CRISPR sites in the ribozymes site is a good idea (FigS4), but the authors should show a downstream application by performing an in vivo experiment on them.

We have now performed knockin experiments at the universal CRISPR sites, showing the expression of an mRFP reporter in *alb* expressing cells (Fig. S4d-e).

-FigS4a, could be nice to show where is the flipped sequence in the OFF version before Cre and Flp activation.

We have now added this information in the schematic of Fig. S4a.

-FigS2b: so the mRNA level is reduced but the gel shows no reduction? This is confusing. Is it because the RT-PCR was saturated? If so, a RT-PCR should be performed with an appropriated number of cycles to not saturate.

The RT-PCR is indeed performed at saturation to show that the mature mRNA in $alb^{HHR/HHR}$ embryos is of wild-type size. We have now included details of this experiment in the Results section (lines 182-184). We have also used the ABE-ultramax base editing method (Qin et al., 2024) to inactivate the ribozyme in the *alb* gene, to show that the pigmentation phenotype is due to ribozyme cleavage rather than splicing defects (Fig. 2g-k).

References

Qin W, Liang F, Lin SJ, Petree C, Huang K, Zhang Y, et al. ABE-ultramax for high-efficiency biallelic adenine base editing in zebrafish. Nat Commun. 2024;15(1):5613.
